# Supplementary material for: Are some individuals generally more behaviorally plastic than others? An experiment with sailfin mollies
Source: PeerJ. 2018 Aug 7;6:e5454. doi: 10.7717/peerj.5454 (PMC6086093; doi:10.7717/peerj.5454)
Supplement: Supplemental Information 2 [file peerj-06-5454-s002.docx]

**SUPPLEMENTARY MATERIAL 1**


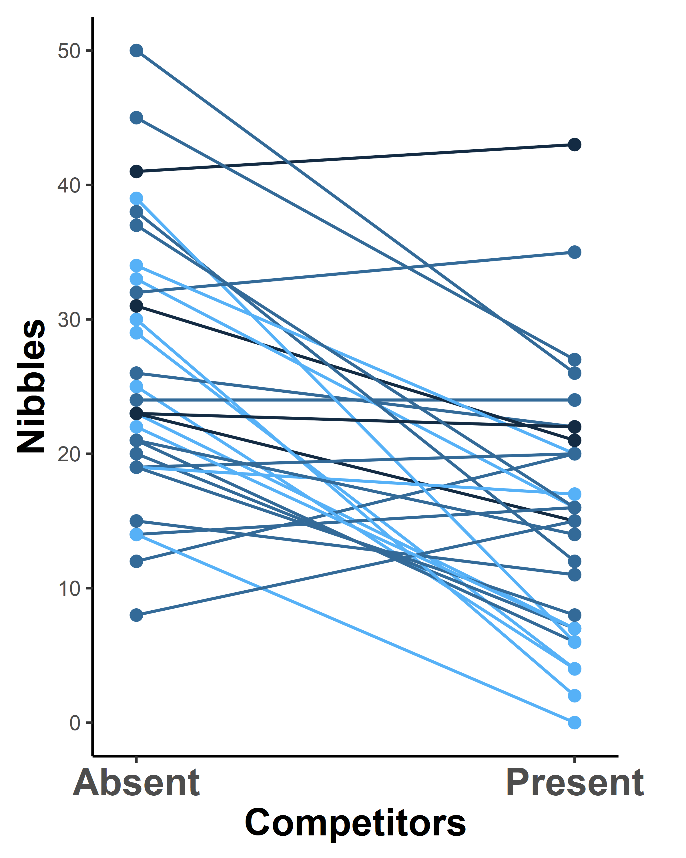
**
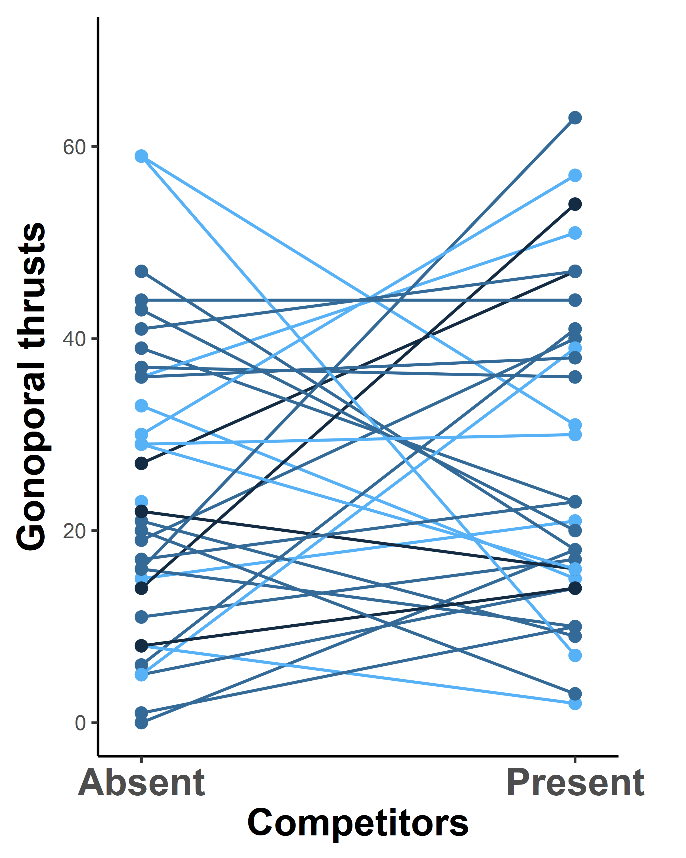
**

**Figure S1. Number of nibbles and gonoporal thrusts by each male when it was tested either in the absence or in the presence of competitors.** Colors indicate the male size category, with lighter colors representing smaller males. None of the behaviors was influenced by the housing conditions prior to the 24h isolation period (LMM controlled for fish identity: nibbles: t_31_=1.000, P=0.325, gonoporal thrusts: t_31_=-0.590, P=0.559). The number of nibbling bites and gonoporal thusts were highly correlated in both conditions (LM: Competitors absent: t_31_=6.816, P<0.001, Competitors present: t_31_:2.329, P=0.027). However, the presence of competitors influenced the number of nibbles (LMM controlled for fish identity: t_32_=-6.084, P<0.001) but not the number of gonoporal thrusts (LMM controlled for fish identity: t_32_=-1.010, P=0.320).
