# Supplementary material for: Are some individuals generally more behaviorally plastic than others? An experiment with sailfin mollies
Source: PeerJ. 2018 Aug 7;6:e5454. doi: 10.7717/peerj.5454 (PMC6086093; doi:10.7717/peerj.5454)
Supplement: Supplemental Information 3 [file peerj-06-5454-s003.docx]

**SUPPLEMENTARY MATERIAL 2**


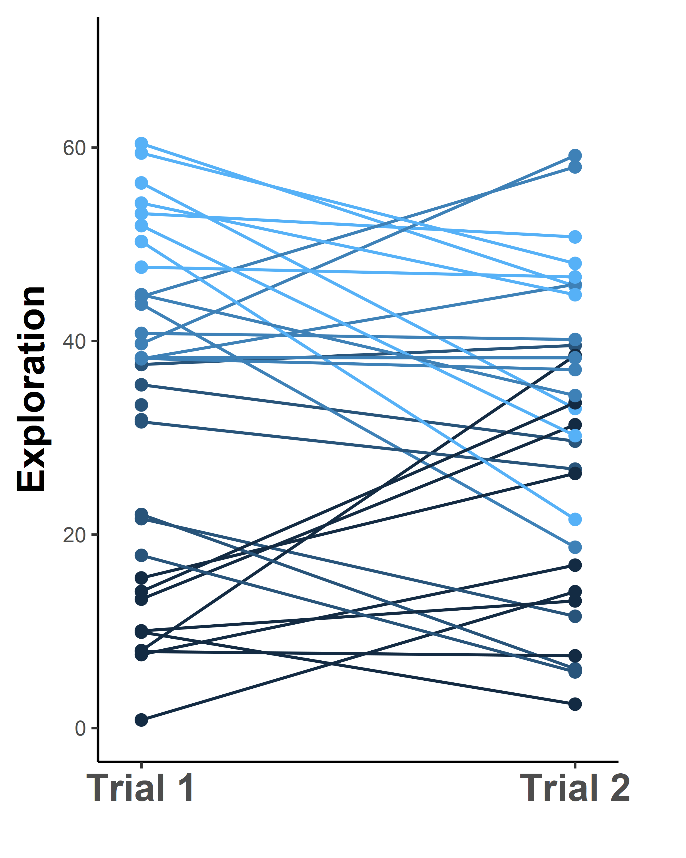

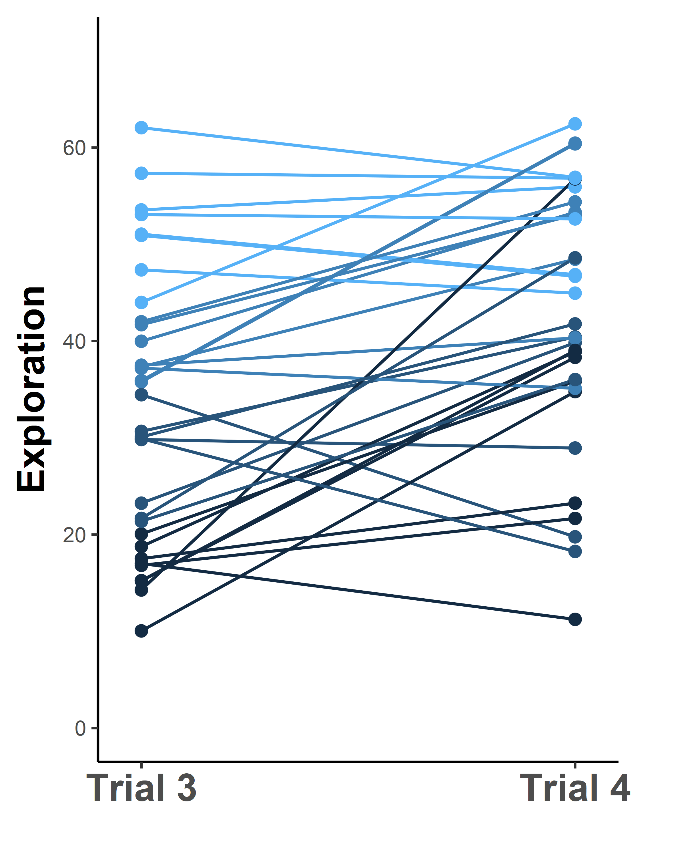

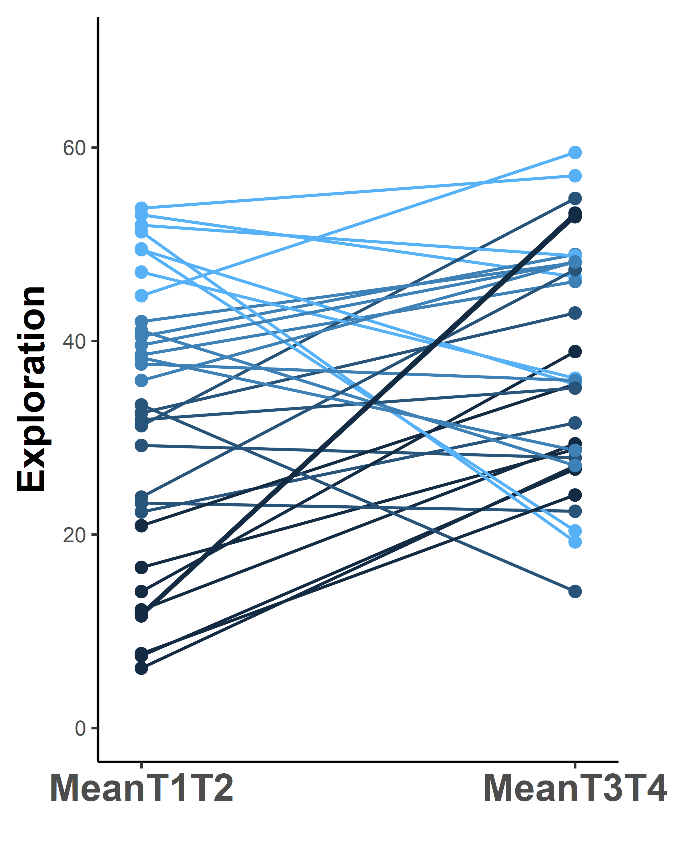


**Figure S2A. Number of different squares visited by the fish during the 5-min observation period when they were either tested alone (i.e. Trials 1 and 2) or with a randomly selected audience (i.e. Trials 3 et 4).** Colors indicate the quantile of personality scores measured during the first exposure to the stimuli, with lighter shades representing individuals with the highest exploratory scores. Individuals significantly altered their exploration tendency across trials (LMM with a continuous variable for trials 1 through 4 and controlled for fish identity: t_96_=3.136, P=0.003). Their behavior also differed on average between the alone and audience treatments (LMM with a fixed factor coding for the treatment and controlled for fish identity: t_96_=2.665, P=0.009). Individuals with maximal scores in both trials/conditions were not removed from the plots (but were removed from the analyses to avoid a ceiling effect).


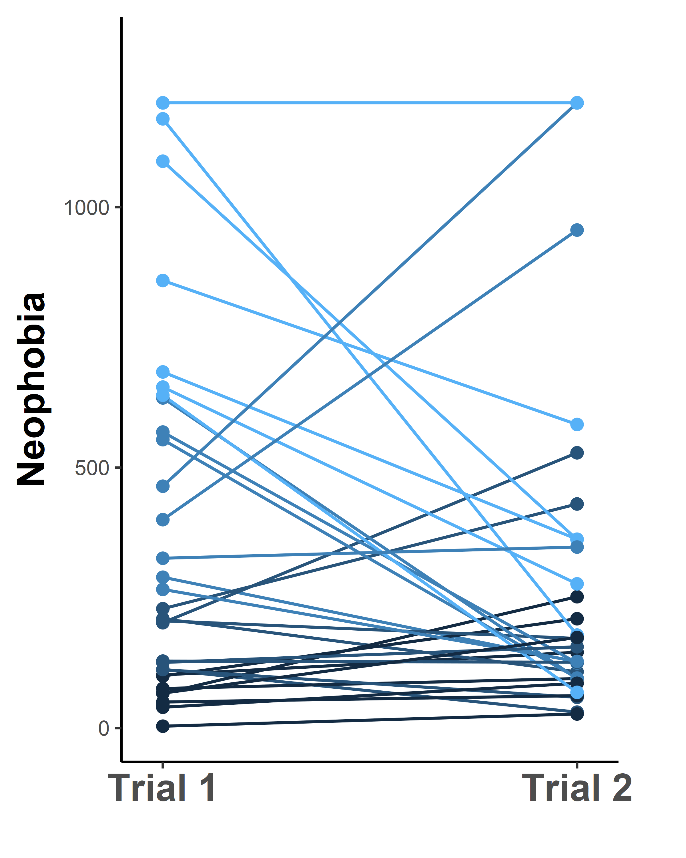

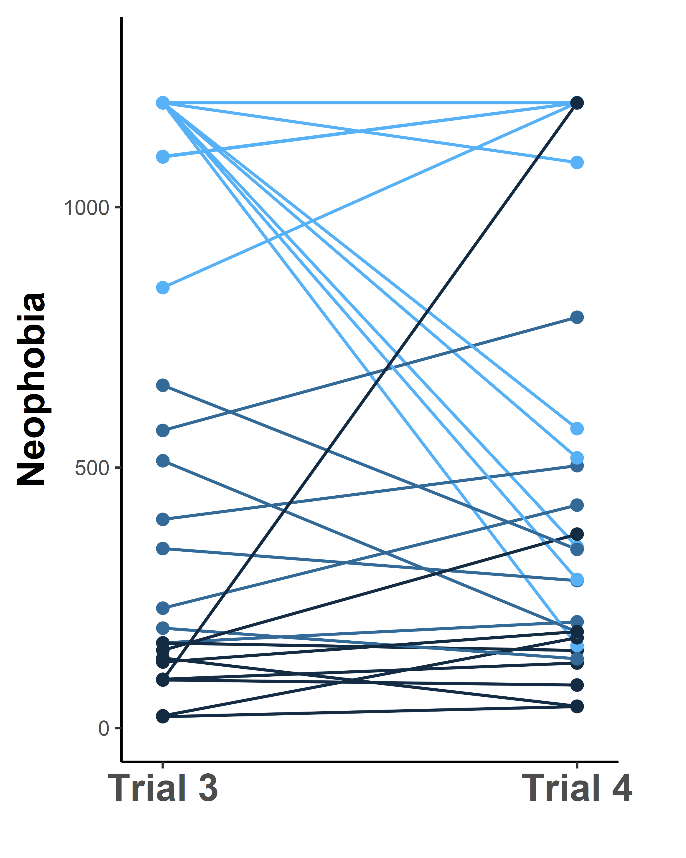

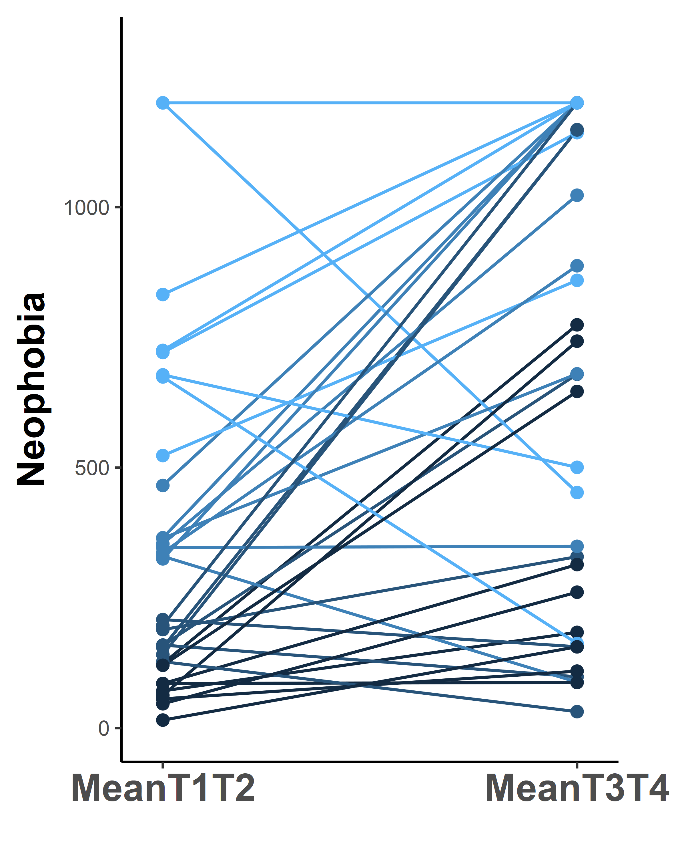


**Figure S2B. Latency times taken by fish when they were tested either alone (i.e. Trials 1 and 2) or with a randomly selected audience (i.e. Trials 3 et 4).** Colors indicate the quantile of the personality scores measured during the first exposure to the stimuli, with lighter shades representing individuals with the highest neophobia scores. Individuals significantly altered their neophobia level across trials (LMM with a continuous variable for trials 1 through 4 and controlled for fish identity: t_96_=3.880, P=0.003). Their behavior also differed on average between the alone and audience treatment (LMM with a fixed factor coding for the treatment and controlled for fish identity: t_98_=5.195, P<0.001). Individuals with maximal scores in both trials/conditions were not removed from the plots (but were removed from the analyses to avoid a ceiling effect).


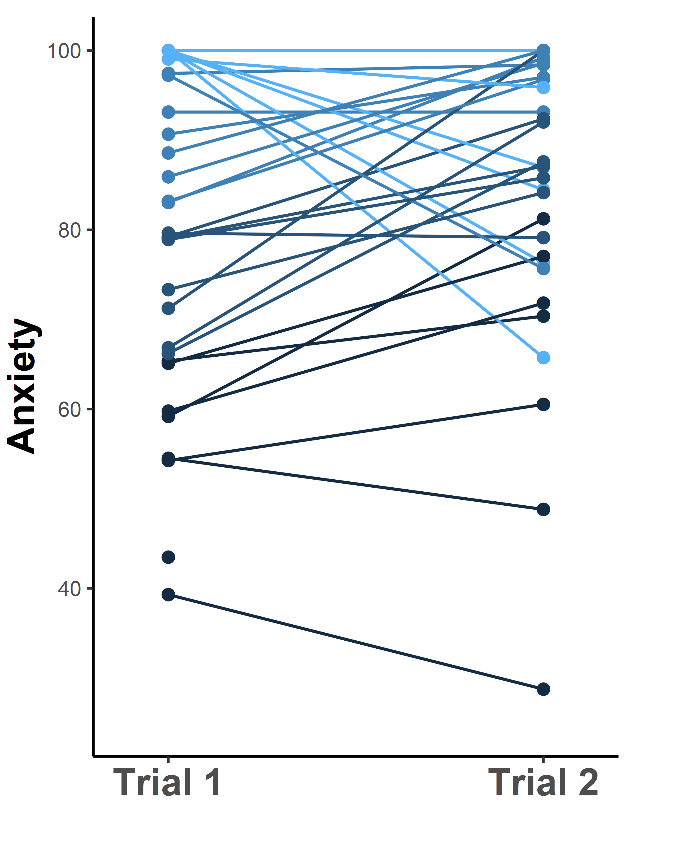


**Figure S2C. Number of squares located at the periphery of the tank hat were visited by the fish without an audience (i.e. Trials 1 and 2).** Colors indicate the quantile of the personality scores measured during the first exposure to the stimuli with lighter shades representing individuals with the highest anxiety scores. Individuals with maximal scores in both trials/conditions were not removed from the plots (but were removed from the analyses to avoid a ceiling effect).
